# Assessment of Folic Acid Supplementation in Pregnant Women by Estimation of Serum Levels of Tetrahydrofolic Acid, Dihydrofolate Reductase, and Homocysteine

**DOI:** 10.1155/2016/1520685

**Published:** 2016-03-15

**Authors:** Manisha Naithani, Vartika Saxena, Anissa Atif Mirza, Ranjeeta Kumari, Kapil Sharma, Jyoti Bharadwaj

**Affiliations:** Department of Biochemistry, All India Institute of Medical Sciences, Virbhadra Road, Rishikesh, Uttarakhand 249 201, India

## Abstract

*Background.* Status of folic acid use in pregnant women of the hilly regions in North India was little known. This study was carried out to assess the folic acid use and estimate folate metabolites in pregnant women of this region.* Materials and Methods*. This cross-sectional study is comprised of 76 pregnant women, whose folic acid supplementation was assessed by a questionnaire and serum levels of homocysteine, tetrahydrofolic acid (THFA), and dihydrofolate reductase (DHFR) were estimated using Enzyme Linked Immunoassays.* Results.* The study data revealed awareness of folic acid use during pregnancy was present in 46.1% and 23.7% were taking folic acid supplements. The study depicted that there was no statistically significant difference between serum levels of THFA and DHFR in pregnant women with and without folic acid supplements (*p* = 0.790). Hyperhomocysteinemia was present in 15.78% of the participants.* Conclusion.* Less awareness about folic acid supplementation and low use of folic acid by pregnant women were observed in this region. Sufficient dietary ingestion may suffice for the escalated requirements in pregnancy, but since this cannot be ensured, hence folic acid supplementation should be made as an integral part of education and reproductive health programs for its better metabolic use, growth, and development of fetus.

## 1. Introduction

Folate is an essential micronutrient which cannot be biosynthesized by human body and must be obtained either from diet or from supplements; it is required for fetal body metabolism, growth, and development in pregnant women. Folate a naturally occurring nutrient is found in foods such as green leafy vegetables, legumes, egg yolk, liver, and citrus fruits. It has role in transmethylation reactions and in de novo biosynthesis of DNA in growing cells. It also serves as a substrate for enzymatic reactions involved in synthesis of amino acid and vitamin metabolism. Folic acid is the synthetic form of folate. Neither folate nor folic acid is metabolically active. Both must be reduced to participate in cellular metabolism. Folic acid on absorption is reduced to tetrahydrofolic acid (THFA) by dihydrofolate reductase (DHFR). THFA has an important role in the metabolism of one carbon groups. Folic acid, in the form 5,6,7,8-tetrahydrofolic acid (THF), is indispensable for single-carbon unit transfer and inters conversion to formyl, methyl, and methylene groups. Thus THFA is labelled as an active form of folic acid. In a study conducted on pregnant women, THFA levels were higher in cord blood when compared to maternal serum, with a probable accumulation of THFA in the fetus [1]. Homocysteine is a thiol compound derived from methionine. It is involved in two main metabolic pathways, namely, the cycle of activated methyl groups which requires folate and vitamin B12 and formation of cystathionine requiring vitamin B6 as cofactor [2]. Elevations in homocysteine may be caused by genetic defects in enzymes involved in its metabolism or by deficiencies in cofactor levels. Homocysteine autooxidizes and damages endothelium increasing cardiovascular risk and increases chances of thrombosis, also linked to Alzheimer's disease and NTD [3]. Hyperhomocysteinemia is a medical condition characterized by very high level of homocysteine in blood; it can be multifactorial, with deficiency of vitamin B12, folic acid, and pyridoxine (B6) all leading to increased homocysteine levels. The folate metabolism represents an interesting model of gene-environment interaction. Methylene tetrahydrofolate reductase (MTHFR) is an important enzyme in folic acid and homocysteine metabolism. The MTHFR gene has many prevalent polymorphisms and these are associated with reduced enzymatic activity and increased plasma homocysteine concentrations [4]. Polymorphism is thus associated with increased risk of NTD. Similarly the enzyme dihydrofolate reductase (DHFR) converts consumed folic acid to THFA. Recently a prevalent polymorphism of the DHFR gene has been described. Studies have suggested that the mutation leads to an increased risk of NTD [5]. Preconception folic acid supplementation is an essential element of reproductive health services. Low maternal folate status has been linked to birth defects such as neural tube defects (NTDs) [6]. Globally each year 3-4 lakhs of infants are born with spina bifida and anencephaly. The prevalence is approximately 1–5 per 1000 live births [7]. In India the overall prevalence has been found to be 4.1 per 1000 by a study [8]. The study conducted in the northern region of India including the Uttarakhand found highest pooled prevalence of NTDs of 7.7 per 1000 total births [9]. This data suggests that neural tube defects contribute to a considerable number of live births and stillbirths in India and makes it imperative to look for the prevention strategies. Supplementation with folic acid protects against NTDs [10, 11]. The efficacy of folic acid supplementation has been demonstrated time and again with the stress on the need of preconceptual intake of folic acid [12, 13].

But the prevention by simple maternal periconceptional intake of folic acid has also been challenged by various studies pointing towards a complex interaction of genetic and environmental factors playing a role in pathogenesis. High incidence of neural tube defects was known from literature, but no study has been conducted on folic acid supplementation in Uttarakhand. Thus the present study was carried out to get insight of awareness of folic acid use and estimate the levels of metabolites of folate metabolism in pregnant women of this region.

## 2. Method and Material

The present study was a clinic based cross-sectional study. It was approved by the ethics committee of the All India Institute of Medical Sciences, Rishikesh. Seventy-six pregnant women attending Primary Health Care Center and Community Health Center Block Doiwala, Dehradun, were included in the study. For the present study pregnant women in first trimester pregnancy were included. The subjects were selected irrespective of folic acid supplementation status. An informed consent was taken from all participants before including them in the study. The subjects taking any medications known to interfere with folate absorption at the time of sampling, using an antifolate medication, or having a history of any disorders or conditions that could interfere with folate absorption were excluded from the study.

A 5 mL of fasting intravenous blood sample was withdrawn from each included study subject, after a short in-person interview including questions related to use of folic acid supplement, dietary pattern, brief history, and measurement of height and weight. The dietary intake was gauged using a modified food questionnaire [14].

The use of folic acid supplements was assessed by asking questions to the participants whether they were taking folic acid supplements or any vitamin pills/supplements that contained folic acid with the dosage. The participants were enquired about recommending person; the starting time and duration of folic acid supplements were also asked about and noted. In response to the questions by the subjects they were grouped as (1) pregnant women not taking folic acid supplements and (2) pregnant women taking folic acid supplements.

The collected blood samples were processed within 4 h of collection. Aliquots were stored at −20°C for batch analysis at the end of the study. Serum samples were analysed for homocysteine, tetrahydrofolate, and dihydrofolate reductase levels. All parameters were analysed using fully automated ELISA analyser and washer (Medicpec Pvt Ltd.). Tetrahydrofolate was measured using competitive inhibition enzyme immunoassay technique kit manufactured by Cloud Clone Corp. Serum homocysteine and dihydrofolate reductase levels were estimated using double antibody sandwich enzyme immune assay by a commercial kit manufactured by Shanghai Qayee Biotechnology Co. Ltd. (Qayee-Bio).

The data was entered into Microsoft Excel 2007. Body Mass Index (BMI) was calculated (normal as 18.5–22.9 Kg/m^2^, overweight and obese as >23 Kg/m^2^). The statistical analysis was performed using the SPSS (version 17.0). The results were expressed as the mean ± SD. Frequency tables and cross tables were made. Pearson's Chi-square test was applied in comparisons of independent and dependent proportions.

## 3. Results

The study conducted for finding out the awareness of folic acid use and its assessment by evaluation of serum levels of folate metabolites in pregnant women at hilly regions of Uttarakhand, India, revealed that the mean age of pregnant women as study participants was 24.37 ± 3.5 years. The study observed that only fifteen (19.7%) participated pregnant women were illiterate and the rest of the participants (80.3%) were literate. Ten out of seventy-six subjects documented the history of abortion (9.2%) or still birth (3.9%), while the rest did not report any event suggestive of bad obstetric history. Surprisingly, a large number of the study subjects, 61 (80.3%) out of 76 pregnancies, were reported for planned pregnancies (Table 1).

Regarding the knowledge of folic acid use, only thirty-five subjects (46.1%) knew about use of folic acid, while the remaining forty-one subjects (53.9%) were unaware of its use. Out of those thirty-five participants knowing about folic acid use, only eighteen (23.7%) subjects were taking folic acid supplements after conception. The 11.8% subjects on folic acid supplements were advised by ANM and 19.7% by the attending doctors and only one participant was using folic acid without being advised by ANM or doctor. Fifty-seven (75%) study participants were not taking any folic acid supplements. However the questions pertained to dietary pattern revealed that all study participants were on normal dietary pattern which included food substances rich in folate.

The serum concentrations of homocysteine, THFA, and DHFR were estimated from the groups with and without use of folic acid supplements (Table 2). By applying independent sample *t*-test there is no statistical significant difference between serum levels of estimated folate metabolites between two groups (*p* = 0.790) (Table 2).

**Figure 1:**
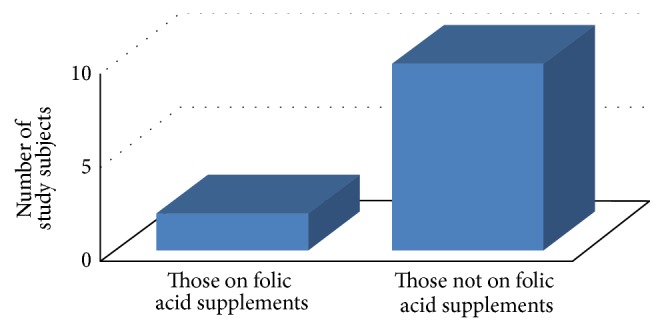
Number of study subjects having hyperhomocysteinemia (>15 micromol/L) in the study.

Hyperhomocysteinemia (more than 15 micromol/L) was noted in 12 (15.78%) of the study participants (Figure 1). Only 2 subjects with use of folic acid exhibited hyperhomocysteinemia (2.63%), but on applying Chi-square test we found there was no statistical significant association between numbers of women with high homocysteine in both groups.

## 4. Discussion

Present study was a step towards assessment of folic acid use, by estimation of serum levels of its metabolites in pregnant women at a Primary Health Center of hilly region in Uttarakhand, India. The study showed higher percentage of participants as literate, with normal BMI, good obstetric history, and planned pregnancies but they had low awareness about of folic acid use during pregnancy. Similar findings were reported in the studies done in other parts of India with low awareness and poor knowledge regarding folic acid deficiency among pregnant women along with poor attitude [15, 16]. Although 75% of study subjects were not on folic acid supplements, there was no statistical significant difference in the serum levels of THFA and DHFR with those subjects who were on folic acid use during pregnancy. This might be due to most of the study participants being on normal diet with increased proportion of food, including folate rich food substances. Such a result can be explained on the basis of known fact that the primary circulating folate vitamer in serum is 5-methyltetrahydrofolic acid, while the actual bioactive form of folate is tetrahydrofolic acid (THFA) which forms a minor component of total serum folate [10]. This was a clinic based cross-sectional study where only motivated women attended and further agreed to take part in the study. Apart from this folate is widely distributed and since most of the women were consuming foods rich in folate, which is apparent by their diet related questions, it is possible that dietary intake sufficed the need of folate. The serum homocysteine levels (micromol/L) were found to be high (more than 15 micromol/L) in 12 (15.78%) studied subjects. Since most of the women were vegetarian (98.02%) hyperhomocysteinemia may be due to moderate vitamin B12 deficiency [17]. Hyperhomocysteinemia can have many explanations and one of the common gene nutrient interactions to be looked at is common polymorphism in the gene of the enzyme 5,10-methylene tetrahydrofolate reductase (MTHFR), known as the MTHFR 677C>T polymorphism, which results in a thermolabile enzyme [18]. But to draw a conclusion about the existing polymorphism as a cause of hyperhomocysteinemia in the present study would not be possible as further genetic studies are required. Also to name only polymorphism as the cause is not possible as many studies have pointed out that the genetic influence which polymorphism has on serum homocysteine levels is attenuated in females in premenopausal age. Hyperhomocysteinemia was less in those taking folic acid (2.63%), but on applying Chi-square test, it was found that there was no significant association between numbers of women with high homocysteine in both groups. Raised homocysteine levels per se have been implicated in complications of pregnancy including preeclampsia, placental abruption, and abortion. Recent studies have shown that the serum homocysteine levels are higher in the women having complicated pregnancy as compared to those without complications [19].

Similar findings have been reported by Katre et al.; their results depicted that increased dose of vitamin B12 but not folic acid was associated with lower plasma total homocysteine concentration in pregnancy [20]. The present study findings have been viewed in light of scarcity of comparable data from India. However, these findings are of importance to practicing obstetricians because the neural tube closure occurs by 28 days of gestation much before the lady knows of pregnancy and attends an antenatal clinic [21]. Many countries have brought policies to enhance folate status of women even before pregnancy, promoting not only supplementation but also use of folate rich foods and fortification [22, 23].

Our study results depicted low information about folic acid use, though efforts have been made to address this issue appropriately by the Government of India that has started promoting the use of folic acid in planned pregnancies during the periconception phase (three months before and three months after conception) for the prevention of NTDs and other congenital anomalies, as a community based intervention, and there are recent studies to advocate the same [24].

The provision of folic acid supplements to women of reproductive age is a key intervention to improve folate status. However, a high degree of compliance can only be achieved by highlighting the affirmative benefits through all-encompassing communication and motivation strategies. There is a need to start preconception clinics as there is high percentage of planned pregnancies [13].

Taking into account the financial constraints the study type was kept cross-sectional and the analysis was performed only for the folic acid metabolites; still the study highlights low information about folic acid use, raised serum homocysteine levels, and bridges the knowledge gap about folic acid metabolites in pregnancy. But in view of the poor use of folic acid supplements in spite of a high rate of planned pregnancies it is recommended that setting up of preconception clinics for motivation strategies not only propagating the benefits of dietary sources but also providing folic acid supplements to women of reproductive age may be a key intervention.

## 5. Conclusion

The study gave us a glimpse into the folic acid metabolism in pregnant women of this region and pointed out inadequacies of health system related to folic acid supplementation. It is noteworthy that homocysteine levels are higher in those not supplemented with folic acid which showed awareness is required to enhance the use of folic acid supplements among pregnant women. Sufficient dietary ingestion may suffice for the escalated requirements in pregnancy, but since this cannot be ensured, hence folic acid supplementation should be made as an integral part of education and reproductive health programs, for an efficient metabolic use, growth, and development of fetus, preventing it from clinical complications. As the present study revealed very important findings, a further research may be conducted with a larger sample size covering wider region, including vitamin B12 to gather a complete picture and for generalization of the findings.

## Figures and Tables

**Table 1:** The descriptive data of pregnant women participating in the study (*N* = 76).

Variable	Frequencies (%)
Age (mean ± SD)	24.37 ± 3.5

Education	
Illiterate	15 (19.7%)
Literate	61 (80.3%)

History of abortion	7 (9.2%)

History of still child birth	3 (3.9%)

Pregnancy planned	61 (80.3%)

Dietary pattern	Vegetarians (98.02%) Green leafy vegetables (89%) Citrus fruit (65%) Eggs (2%)

BMI	Normal in 68 (89.47%) Overweight and obese in 8 (10.53%)

**Table 2:** Serum levels of homocysteine, tetrahydrofolate, and dihydrofolate reductase in pregnant women (*N* = 76).

Biochemical parameters assessed	Pregnant women with folic acid supplementation (*n* = 18)	Pregnant women without folic acid supplementation (*n* = 57)	*p* values
Serum homocysteine levels (micromol/L)	11.05 ± 3.773	11.03 ± 3.989	0.981
Serum tetrahydrofolate levels (nmol/L)	0.99 ± 1.44	1.10 ± 1.50	0.790
Serum dihydrofolate reductase levels (pg/mL)	1261.87 ± 489.17	1298.08 ± 755.69	0.849
